# Homozygous Mutation in the FANCD2 Gene Observed in a Saudi Male Infant with Severe Ambiguous Genitalia

**DOI:** 10.1155/2021/6686312

**Published:** 2021-07-16

**Authors:** Aida Al Jabri, Nusaybah Al Naim, Abeer Al Dossari

**Affiliations:** ^1^Department of Pediatrics, King Abdulaziz Hospital, Ministry of the National Guard-Health Affairs, King Abdullah International Medical Research Center, King Saud Bin Abdulaziz University for Health Sciences, Al-Ahasa, Saudi Arabia; ^2^Department of Pediatrics, King Abdulaziz Hospital, Ministry of National Guard-Health Affairs, Al-Ahasa, Saudi Arabia

## Abstract

Fanconi anemia (FA) is a rare autosomal recessive inherited disease caused by gene mutations that are primarily involved in the response to or repair of DNA damage. FA characterizes by multiple congenital abnormalities and malformations including growth retardation, renal agenesis, absence of radial bones and thumbs as well, progressive bone marrow failure, irregular skin pigmentation patterns, and increased susceptibility to cancer. FANCD2 gene mutation is believed to be one of the causative mutations in Fanconi anemia, and despite many case reports that link the FANC gene mutation to multiple congenital anomalies and disease, there is no case report found to link it with genitalia abnormalities. In our paper, we report a male Saudi infant who presented to the endocrine clinic at the age of 9 months with severe ambiguous genitalia and found that he carries a homozygous variant mutation in the FANCD2 gene and we face a challenge to treat this patient since there was no previous similar case.

## 1. Introduction

Fanconi anemia (FA) is a rare autosomal recessive inherited disease caused by gene mutations that are primarily involved in response to or repair of DNA damage. Congenital malformations, progressive bone marrow failure, irregular skin pigmentation patterns, and cancer susceptibility also characterize the disorder [1].

With the exception of FANCB, which is located on the X chromosome, there are more than 15 FANC gene mutations associated with Fanconi anemia. All other FANC genes are autosomal recessive inheritance [2].

There are multiple congenital abnormalities such as growth retardation, renal agenesis, absence of radial bones and thumbs, and radial deviation of the hands that are also correlated with the FANCD2 gene mutation, which is believed to be one of the causative mutations in Fanconi anemia [3], nasopharyngeal carcinoma [4], hepatocellular carcinoma [5], and interstitial nephritis [6].

Although there were many case reports that link the FANC gene mutation to multiple congenital anomalies and diseases, no case report was found to link it with genitalia abnormalities.

There is some evidence in an animal study that FANCB is essential in the male germline and regulates H3K9 methylation on the sex chromosomes during meiosis [7]; also, it was found in one case report that FANCM mutation was linked to primary ovarian insufficiency [8].

In our case, we found that FANCD2 gene mutation was linked to ambiguous genitalia in males. Genital ambiguity considers one of the most important medical emergencies in pediatrics, not only because it is linked to salt wasting and electrolyte imbalance but also because it has long-term psychosocial effects for patients and their families.

The incidence of disorders of sex development (DSD) is not fully known. In 2000, Fausto-Sterling suggested that it corresponds to 1.7% of live births [9].

In one small study conducted in our region (ALHAFOUF) that included 41 newborn infants, it has been found that, in 46, XX karyotype patients, congenital adrenal hyperplasia followed by general malformation disorder was the most common cause of ambiguous genitalia in more than two-thirds of patients while in XY karyotype patients, testosterone pathway biosynthetic defects were the most common cause even in conjunction with a generalized malformation disorder [10].

## 2. Case Presentation

The case of a 30-month-old Saudi boy from Al-Ahsa is presented. He was born at term with ambiguous genitalia and intrauterine growth restriction (IUGR) with a birth weight of 1.6 kg by normal delivery at a local hospital. He was the fourth child in the family, and his parents are first cousins. There was no family history of ambiguous genitalia, neonatal death, or skeletal abnormalities, and the pregnancy was unremarkable.

After birth, the patient was admitted to the intermediate care nursery (ICN) for 18 days as a neonate with ambiguous genitalia for investigation. He was seen by an endocrinologist at that time, and he found that the baby was not dysmorphic with no midline defect, both gonads were palpated in the well-developed scrotum with severe hypospadias, the Stretched Penile Length (SPL) of 2 cm, and the anus was sited normally; otherwise, the examination was normal. Pelvic ultrasound (US) showed small testes with no female internal genitalia, and karyotype revealed a 46 XY. The impression at that time was a male neonate with severe hypospadias and no disorder of sex development (DSD) and to be seen by the urology team for hypospadias correction.

At the age of 9 months, he was seen by a urologist and his impression was clear ambiguous, so he was referred to another endocrinologist for further investigation and management.

In our clinic, when we saw him and on physical examination (at the age of 9 months), he was pale, with failure to thrive (FTT) and both height and weight below the −2 SD. He was not dysmorphic with no organomegaly. Genitalia exam was ambiguous with severe penile chordee, small phallus with a stretched penile length (SPL) of 2 cm, perineal hypospadias, bifid scrotum, and bilateral small testes. Laboratory investigation performed includes an HCG stimulation test. The injection of 500 international units (IUs) was given over 3 days. Testosterone, dihydrotestosterone, and androstenedione were collected before the first injection and 24 hours after the last one. The results were normal with a testosterone-to-dihydrotestosterone ratio of 10.1 and testosterone-to-androstenedione ratio of 23.

So, we sent a gene to clarify the causes of the ambiguous genitalia which showed homozygous variant (c.2605 + 1G>A p.) mutation in the FANCD2 gene (OMIM: 613984), which was considered as the first homozygous mutation of this gene.

To complete our investigation, electrolytes were normal, and complete blood count (CBC) revealed an iron deficiency anemia (IDA) with hemoglobin of 8.3 mg/dL and a low mean corpuscular volume (MCV) and mean corpuscular hemoglobin (MCH) of 56.8 fL and 18.2 fL, respectively. The red cell distribution width (RDW) was 16%, and the platelet level was normal. A skeletal survey was performed, and it was normal.

**Figure 1:**
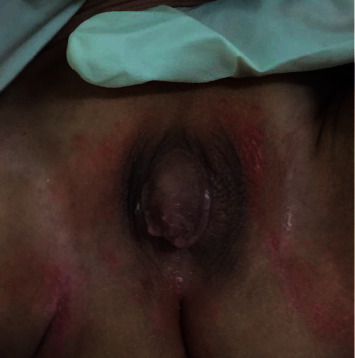
The genitalia before treatment.

**Figure 2:**
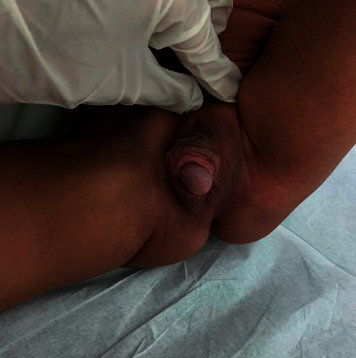
The genitalia after 3 doses of testosterone injection.

So, since this is the first case with such variant and abnormal genitalia and with no previous similar case, we planned to challenge him with testosterone injection; he received a total of 4 doses of 50 mg intramuscular (IM) injection every 4 weeks; in Figure 1, his genitalia before treatment is shown, and Figure 2 shows his genitalia after the last dose.

Genitalia examination after the last dose showed much improvement in the length of his penis with an SPL of 3.5 cm and palpable testes in the scrotum with severe hypospadias. Also, he was treated for the FTT and IDA.

One month after his last injection, he showed further improvement with an SPL of 4.5 cm, and surgical correction for the hypospadias was performed over stages.

## 3. Discussion and Conclusions

Fanconi anemia (FA) is an autosomal recessive genetic disorder characterized by progressive bone marrow failure, multiple congenital anomalies, and an increased risk of cancer, particularly acute myeloid leukemia [11].

The cellular phenotype is characterized by chromosomal instability and hypersensitivity to DNA interstrand crosslinking (ICL) agents such as mitomycin C (MMC), diepoxybutane, and cisplatin. There are considered to be at least eight complementation groups (FA-A, B, C, D1, D2, E, F, and G) [11].

FANCD2 is an evolutionary gene of Fanconi anemia (FA), and it is considered one of the most important genes that play a key role in reactions to DNA double-strand type damage [12].

Congenital malformations are common, and hematological symptoms occur faster and rapidly progressive in those patients with FANCD2 gene mutation rather than the other patients [12].

Although there were many case reports linking the FANC gene mutation to multiple congenital anomalies and diseases, no case report was found to link this disease with genitalia ambiguity. The phenotype of FA in children has been extensively described before including endocrine and nonendocrine manifestations; male genitalia abnormalities have a high incidence in those patients with FA, and it is around 20% of the FA clinical manifestations. It includes hypoplastic gonads, hypogenitalia, undescended testes, micropenis, cryptorchidism, hypospadias, and hypo-/azoospermia with infertility. Females may also have underdeveloped genitalia and malpositioned uterus with small ovaries [13–16].

There is some evidence in an animal study that FANCB is essential in the male germline and regulates H3K9 methylation on the sex chromosomes during meiosis [7]; also, it was found in one case report that FANCM mutation was linked to primary ovarian insufficiency [8].

In our case, we found that FANCD2 gene mutation was linked to ambiguous genitalia in Saudi male infants.

There are a lot of varieties of disorders under the term ambiguous genitalia which are labeled as DSDs or disorders of sex development. DSDs are categorized as congenital disorders in which chromosomal, gonadal, or anatomical sex development is abnormal, and they include a comprehensive variety of metabolic and anatomical deficiencies and variations which can cause atypical genital appearance, and if left untreated, they can cause distress that is emotional and psychological [17].

Through a well-choreographed, well-coordinated, and localized expression of gene products, a normal sex differentiation and development progress can be developed; furthermore, the gonads, as well as internal and external genital structures all, originating from the same bipotential embryologic tissues are important to be understood or realized [17].

The first migration of bipotential primordial germ cells from the yolk sac endoderm to the urogenital ridges leads to the undifferentiated gonad beginning at 4–6 weeks [17].

Even though, histologically, changes are well underway, the male and female fetuses could not be distinguished in the first 7 weeks of pregnancy which is considered as an indifferent stage. Later on, a switch could occur that signals the determination of the sex and leads to the initiation of testicular or ovarian gonadal differentiation [17].

In normal sexual differentiation, several genes are involved. The molecular events of testis formation are triggered by the sex-determining region on the Y chromosome (SRY) [18]. Congenital adrenal hyperplasia (CAH) is one of the most causes of ambiguous genitalia. Wide-ranging phenotypes from a salt-wasting adrenal crisis in the infant to virilization in young females delayed puberty in adolescents, and even polycystic ovary syndrome- (PCOS-) like symptoms (i.e., acne, irregular menses, hirsutism, and infertility) in young women are as a result of a collection of different adrenal steroid biosynthetic disorders. In any virilized infant with nonpalpable gonads, CAH should be the primary consideration [17].

The treatment of the abnormal genital is considered a challenge for the pediatrician and endocrinologist providing care. It is crucial to determine the cause and treat it as soon as possible to minimize the medical, psychological, and social complications. It can be a mild form that required only testosterone injection to surgical sex reverse in the severe form [19, 20].

In our case, the treatment was a challenge since no case report before describes the same manifestation; however, we succeeded in treating him with testosterone injection, although we had to follow him up for further pubertal assessment.

## Data Availability

The data used to support the findings of this study are available from the corresponding author upon request.
